# Preoperative Clinical Characteristics Predict Recurrent Laryngeal Nerve Lymph Node Metastasis and Overall Survival in Esophageal Squamous Cell Carcinoma: A Retrospective Study With External Validation

**DOI:** 10.3389/fonc.2022.859952

**Published:** 2022-03-31

**Authors:** Hao-Ji Yan, Wen-Jun Mao, Rui-Xuan Yu, Kai-Yuan Jiang, Heng Huang, Zheng-Dong Zong, Qin-Chun Qian, Xiao-Guang Guo, Hong-Ying Wen, Dong Tian

**Affiliations:** ^1^ Department of Cardiothoracic Surgery, Affiliated Hospital of North Sichuan Medical College, Nanchong, China; ^2^ College of Medical Imaging, North Sichuan Medical College, Nanchong, China; ^3^ Department of Cardiothoracic Surgery, The Affiliated Wuxi People’s Hospital of Nanjing Medical University, Wuxi, China; ^4^ Department of Thoracic Oncology, Cancer Center, West China Hospital, Sichuan University, Chengdu, China; ^5^ College of Clinical Medicine, North Sichuan Medical College, Nanchong, China; ^6^ Department of Pathology, Nanchong Central Hospital, Nanchong, China

**Keywords:** esophageal squamous cell carcinoma, recurrent laryngeal nerve, lymph node metastasis, nomogram, prognosis

## Abstract

**Background:**

Recurrent laryngeal nerve (RLN) lymph node metastasis (LNM) is not rare in patients with esophageal squamous cell carcinoma (ESCC). We aimed to develop and externally validate a preoperative nomogram using clinical characteristics to predict RLN LNM in patients with ESCC and evaluate its prognostic value.

**Methods:**

A total of 430 patients with ESCC who underwent esophagectomy with lymphadenectomy of RLN LNs at two centers between May 2015 and June 2019 were reviewed and divided into training (center 1, n = 283) and external validation cohorts (center 2, n = 147). Independent risk factors for RLN LNM were determined by multivariate logistic regression, and a nomogram was developed. The performance of the nomogram was assessed in terms of discrimination, calibration, clinical usefulness, and prognostic value. The nomogram was internally validated by the bootstrap method and externally validated by the external validation cohort.

**Results:**

Multivariate analysis indicated that clinical T stage (*P* <0.001), endoscopic tumor length (*P* = 0.003), bioptic tumor differentiation (*P* = 0.004), and preoperative carcinoembryonic antigen level (*P* = 0.001) were significantly associated with RLN LNM. The nomogram had good discrimination with the area under the curve of 0.770 and 0.832 after internal and external validations. The calibration curves and decision curve analysis confirmed the good calibration and clinical usefulness of this model. High-risk of RLN LNM predicted by the nomogram was associated with worse overall survival in the external validation cohort (*P* <0.001).

**Conclusion:**

A nomogram developed by preoperative clinical characteristics demonstrated a good performance to predict RLN LNM and prognosis for patients with ESCC.

## Introduction

Esophageal carcinoma is a common malignant obstructive esophageal disease with an incidence and mortality of 604 and 544 thousand worldwide and ranks seventh and sixth in terms of incidence and mortality, respectively (1). It is histologically classified into esophageal adenocarcinoma, esophageal squamous cell carcinoma (ESCC), and other subtypes, whereas greater than 90% of esophageal cancer cases are ESCC in Asia countries (2). Although current treatments for ESCC can acquire favorable outcomes in given conditions, the 5-year survival rate is only approximately 29.7% due to lymph node metastasis (LNM) being affected at a rate of approximately 50% (3).

The recurrent laryngeal nerve (RLN) lymph nodes (LNs) are the crucial indicator during esophagectomy, which may decide the necessity of cervical lymphadenectomy in some previous studies (4, 5). The RLN LNM in patients with ESCC is frequently observed at a rate of 18–63% in the previous studies (6–13). Routine esophagectomy with three-field lymph node resection for all ESCC patients is still controversial due to the balance of complication and survival (9, 14). Additionally, the status of RLN LNs was demonstrated to be closely associated with prognosis (15, 16). Hence, the evaluation of the status of RLN LNs using an accurate prediction model before an operation could be valuable to conduct the optimal treatment and improve the prognosis for ESCC patients.

Previous studies reported the risk factors for RLN LNM in esophageal cancer, but the predictive value is limited for a single risk factor (4, 17, 18). Although several reports of a prediction model is based on clinicopathological characteristics for RLN LNM, the performance of a preoperative prediction model needs further evaluation (6, 19, 20). This retrospective study was conducted to assess the preoperative clinical characteristics of ESCC patients and to develop and validate a preoperative nomogram for predicting RLN LNM. Furthermore, we first evaluated the prognostic value of the nomogram for overall survival.

## Methods

### Patients

**Figure 1:**
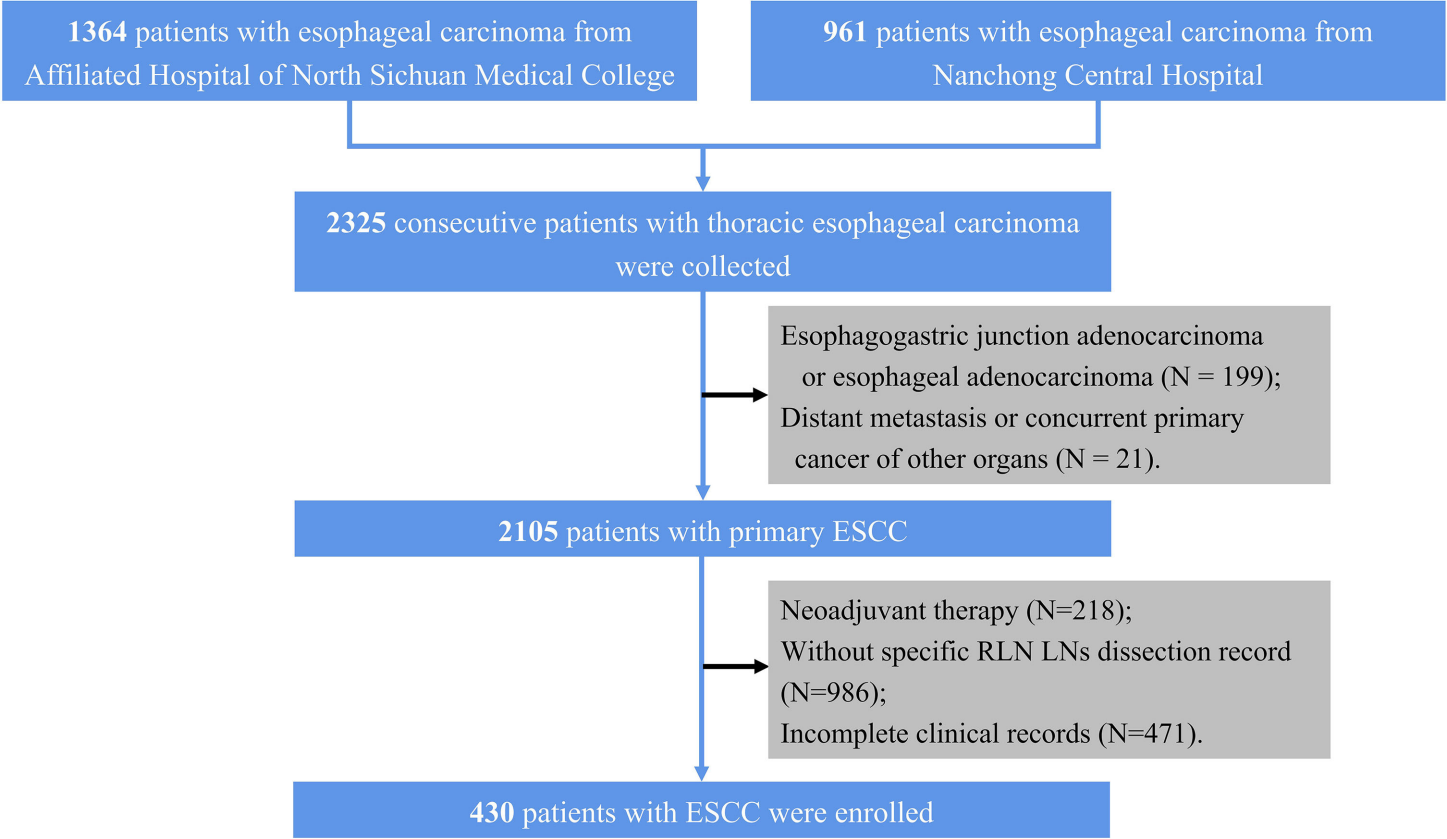
Flow chart of patient enrollment. ESCC, esophageal squamous cell carcinoma.

Patients underwent curative resection for esophageal carcinoma at the Affiliated Hospital of North Sichuan Medical College and the Nanchong Central Hospital and their specimens were collected between May 2015 and June 2019. Patients from the Affiliated Hospital of North Sichuan Medical College (center 1) were set as a training cohort, while patients from the Nanchong Central Hospital (center 2) were assigned to an external validation cohort. The inclusion criteria are as follows. (1) primary ESCC, (2) McKeown esophagectomy with specific RLN LNs dissection record, (3) complete resection (R0 resection), and (4) detailed preoperative clinical data were available. In our center, the RLN LNs may categorize into 2 or 4 groups; hence, we only included patients with specific RLN LNs dissection records. Initially, 2,325 consecutive patients with thoracic esophageal carcinoma were collected. We excluded 199 patients with esophagogastric junction adenocarcinoma or esophageal adenocarcinoma, 21 with distant metastasis or concurrent primary cancer of other organs, 218 who underwent neoadjuvant therapy, 986 without specific RLN LNs dissection record, 471 with incomplete clinical records; hence, a total of 430 patients (283 from center 1 and 147 from center 2) were included in this study (**Figure 1**). The work has been reported in line with the STROCSS criteria (21). The Ethics Committees and Review Board of the Affiliated Hospital of North Sichuan Medical College (No. 2020ER181-1) approved this study, and the need for patient consent was waived.

Data were collected from esophagogastroduodenoscopy, bioptic pathological section, contrast esophagography, endoscopic ultrasonography, neck and abdominal ultrasonography, enhanced computed tomography (CT), and preoperative hematological examination prior to surgery. The following characteristics were reviewed: age, sex, endoscopic tumor length, bioptic tumor differentiation, tumor location, clinical T stage, hemoglobin, neutrophil, preoperative carcinoembryonic antigen (CEA), low-density lipoprotein cholesterol (LDL-C), and high-density lipoprotein cholesterol (HDL-C) levels, surgical approach, RLN LNM status, overall survival. Endoscopic tumor length was defined as the length between the proximal and distal margins of the tumor. The bioptic tumor differentiation was defined based on the cellular differentiation degree in the preoperative bioptic pathological section. The interval of follow-up after surgery was every 1–6 months, and a CT scan was conducted annually. The last follow-up visit was in March 2021.

### Surgery and Staging

McKeown esophagectomy (thoracotomy/video-assisted thoracic surgery [VATS]) was performed in all patients. In both centers, VATS was preferred, while the thoracotomy was used in a relatively large tumor. Meanwhile, if the VATS failed, the surgery will converse to thoracotomy. The esophagectomy was performed by senior doctors. The classification of surgically dissected LNs was in accordance with the definition of regional LNs. All biopsy specimens under esophagogastroduodenoscopy were examined. An experienced pathologist reevaluated LNs to determine the grades of preoperative tumor differentiation and status of RLN LNs. The clinical T stage was mainly determined by the preoperative contrast-enhanced CT image, neck and abdominal ultrasonography following the ESCC TNM classification criteria of the 8th edition American Joint Committee on Cancer (AJCC) & Union for International Cancer Control (UICC) (22).

### Development and Validation of a Nomogram

**Figure 2:**
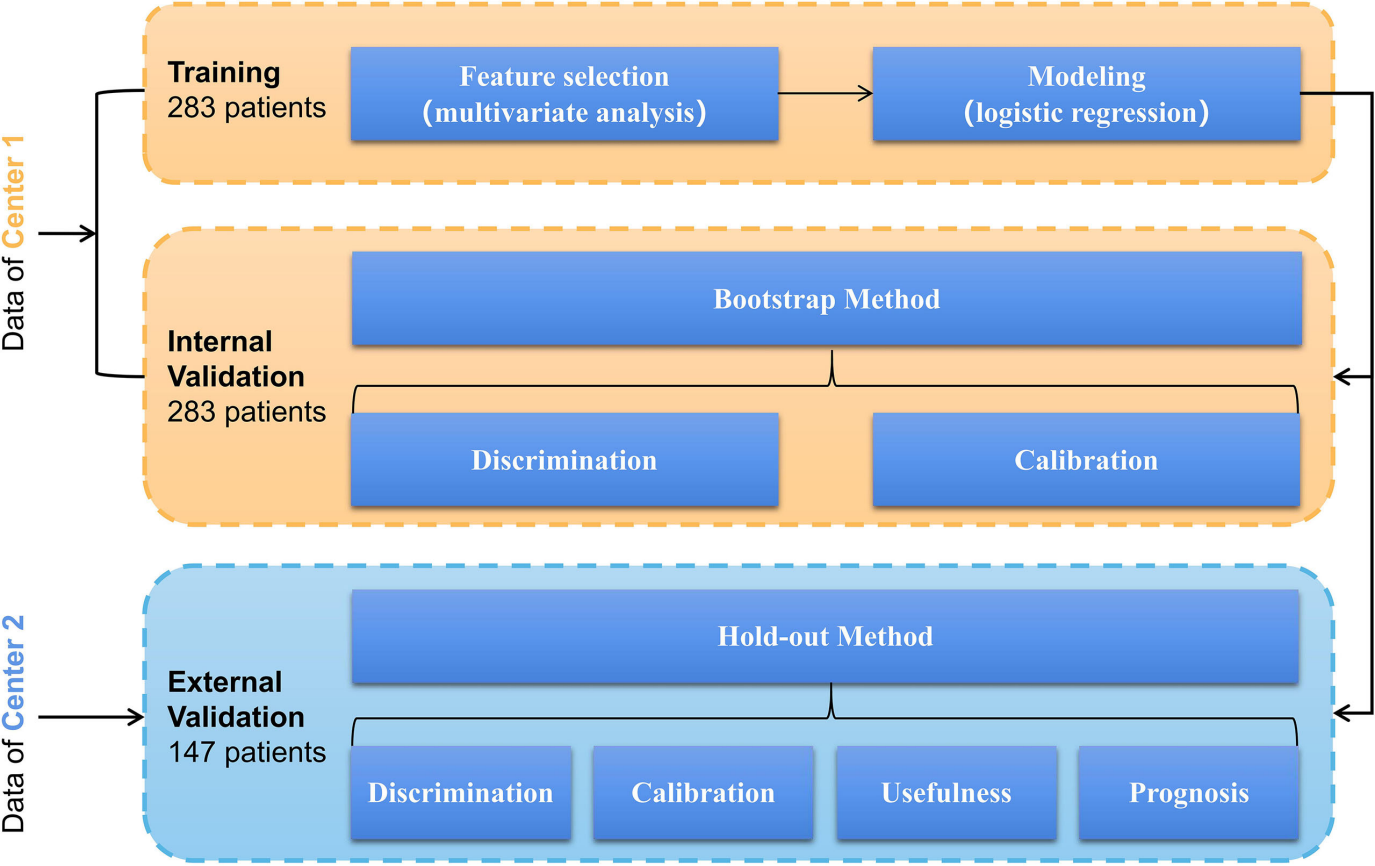
Model’s development and validation based on logistic regression involving a training cohort (283 patients in center 1) and an external validation cohort (147 patients in center 2).

Univariate logistic regression was used to screen factors associated with RLN LNM based on the training cohort. Multivariate logistic regression only included factors with a *P <*0.05 in univariate logistic regression. A preoperative prediction model of RLN LNM was developed by logistic regression using independent risk factors, and a nomogram was established accordingly to visualize the model. The area under the curve (AUC) of the receiver operating characteristic (ROC) curve was used to assess the discrimination of this nomogram. The calibration of the model was assessed by Brier score and calibration curve. Decision curve analysis (DCA) was performed to evaluate the efficiency of this nomogram. Model performance was internally validated by the bootstrap method with 1,000 repetitions and externally validated by the hold-out method (external validation cohort). The prognostic value of the nomogram was evaluated in the external validation cohort only. According to the predicted value of the nomogram, patients were divided into high-risk of RLN LNM and low-risk of RLN LNM by an optimal threshold. Survival curves of the two groups were plotted by the Kaplan–Meier method and compared using the log-rank test. The prognostic values of the RLN LNM and the predicted RLN LNM risk were assessed using multivariable Cox regression adjusting for other factors that did not include the modeling factors to avoid multicollinearity. The detailed process of the development and validation of the model is shown in **Figure 2**.

### Statistical Analysis

Demographic and clinical data were included in the statistical analysis using R (version 3.6.3, www.r-project.org). The continuous and categorical variables were expressed in terms of the mean ± standard deviation and frequencies with proportions. Chi-square tests and Student’s t-tests were conducted to compare the differences in continuous and categorical variables between the training and external validation cohorts. Odds ratios (ORs) and hazard ratios (HRs) with 95% confidence intervals (95% CIs) were calculated. *P <*0.05 was deemed statistically significant.

## Results

### Clinical Characteristics

A total of 430 patients with 306 men and 124 women were enrolled, which predominantly are patients with age ≥60 years (n = 317) (**Supplementary Table 1**). Most patients received VATS with 349 cases, compared to thoracotomy with 81 cases. In the entire cohort, the endoscopic tumor length (*P <*0.001), bioptic tumor differentiation (*P <*0.001), clinical T stage (*P <*0.001), and preoperative CEA (*P <*0.001) in the RLN LNM-positive group were significantly different from those in the RLN LNM-negative group. Patients from center 1 were assigned to training cohort (n = 283) while from center 2 assigned to external validation cohort (n = 147). The clinical characteristics of patients with ESCC in two cohorts are summarized in **Table 1**. The rates of RLN LNM in the entire cohort, training cohort and external validation cohort were 28.8% (124 of 430), 29.0% (82 of 283), and 28.6% (42 of 147), respectively. Endoscopic tumor length (*P* = 0.034), tumor location (*P* = 0.024), clinical T stage (*P <*0.001), hemoglobin (*P* = 0.001), preoperative CEA (*P <*0.001), and LDL-C (*P <*0.001) were significantly different between the training and external validation cohorts. The median follow-up time is 45 months (range: 3–80 months) in the external validation cohort.

**Table 1:** Clinical characteristics of esophageal squamous cell carcinoma patients.

Characteristics	Training cohort (n = 283)	External validation cohort (n = 147)	P-value
Age			0.193
<60 y	80 (28.2%)	33 (22.4%)	
≥60 y	203 (71.8%)	114 (77.6%)	
Sex			0.418
Male	205 (72.4%)	101 (68.7%)	
Female	78 (27.6%)	46 (31.3%)	
Endoscopic tumor length			0.034*
<3 cm	108 (38.1%)	41 (27.8%)	
≥3 cm	175 (61.9%)	106 (72.2%)	
Tumor location			0.024*
Upper	45 (15.9%)	33 (22.4%)	
Middle	154 (54.5%)	87 (59.2%)	
Lower	84 (29.6%)	27 (18.4%)	
Bioptic tumor differentiation			0.397
G1	121 (42.8%)	56 (38.1%)	
G2	129 (45.5%)	77 (52.4%)	
G3	33 (11.7%)	14 (9.5%)	
Clinical T stage^†^			<0.001*
T1/T2	142 (50.2%)	47 (32.0%)	
T3/T4	141 (49.8%)	100 (68.0%)	
Hemoglobin (g/dl)	128.95 ± 14.90	133.91 ± 15.60	0.001*
Neutrophil (10^9^/L)	3.91 ± 2.23	4.25 ± 1.63	0.106
Preoperative CEA (ng/ml)	4.86 ± 1.66	5.55 ± 1.27	<0.001*
HDL-C (mmol/L)	1.30 ± 0.32	1.34 ± 0.37	0.251
LDL-C (mmol/L)	2.82 ± 0.67	3.17 ± 0.76	<0.001*

*P <0.05.

^†^The 8th edition of the UICC and AJCC cancer staging system.

CEA, carcinoembryonic antigen; HDL-C, high density lipoprotein-cholesterol; LDL-C, low density lipoprotein-cholesterol.

### Independent Risk Factors

**Figure 3:**
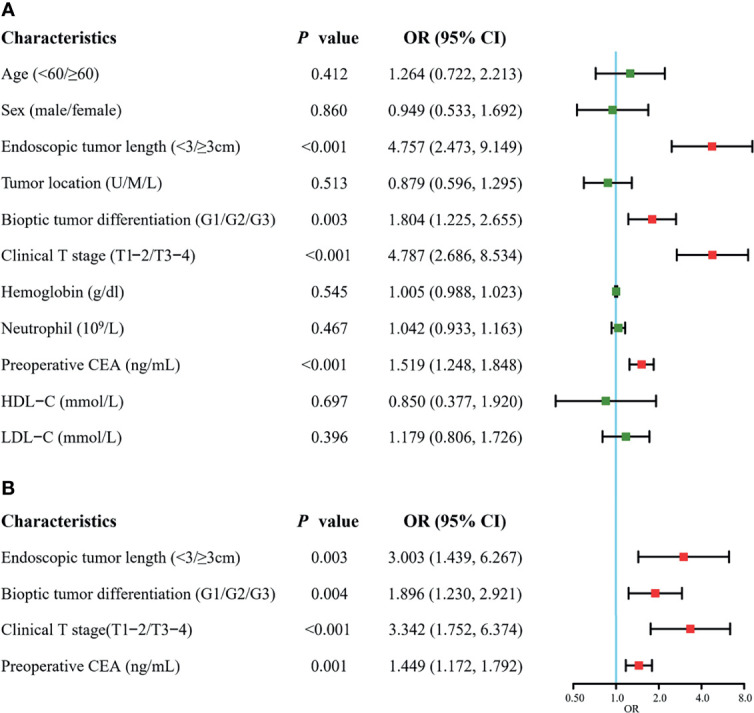
Forest plots of univariate **(A)** and multivariate **(B)** analysis for recurrent laryngeal nerve lymph node metastasis in the training cohort. CEA, carcinoembryonic antigen; HDL-C, high-density lipoprotein-cholesterol; LDL-C, low-density lipoprotein-cholesterol; U, upper; M, middle; L, lower. The clinical T stage and bioptic tumor differentiation were referenced by the 8th edition of the UICC and AJCC cancer staging system.

Forest plots of univariate and multivariate analyses are displayed in **Figure 3**. Univariate analysis indicated that the factors related to RLN LNM included endoscopic tumor length, bioptic tumor differentiation, clinical T stage, and preoperative CEA. No statistically significant differences in age, sex, tumor location, HDL-C, LDL-C, neutrophils or hemoglobin were noted (*P >*0.05). The multivariate analysis revealed that endoscopic tumor length (OR = 3.003, 95% CI, 1.439–6.267, *P* = 0.003), clinical T stage (OR = 3.342, 95% CI, 1.752–6.374, *P <*0.001), bioptic tumor differentiation (OR = 1.896, 95% CI, 1.230–2.921, *P* = 0.004), and preoperative CEA (OR = 1.449, 95% CI, 1.172–1.792, *P* = 0.001) were independent risk factors for RLN LNM.

### Development and Validation of a Nomogram

**Figure 4:**
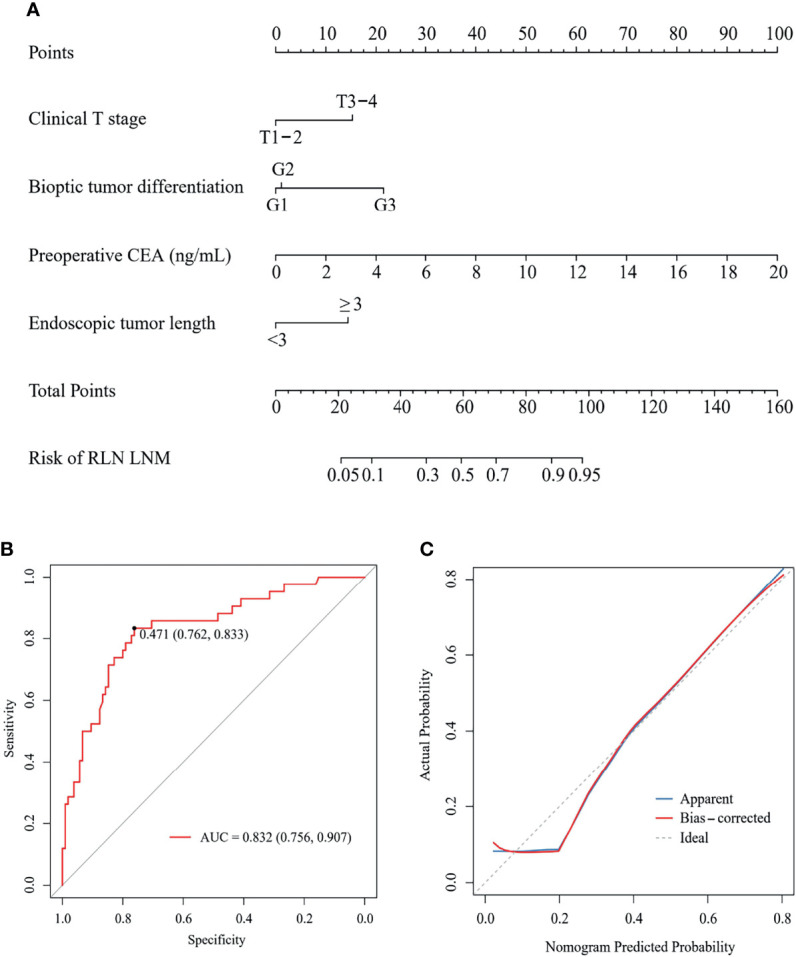
A nomogram **(A)** to predict the risk of RLN LNM and its ROC curve **(B)** and calibration curve **(C)** in external validation. CEA, carcinoembryonic antigen; RLN LNM, recurrent laryngeal nerve lymph node metastasis; ROC, the receiver operating characteristic.

**Figure 5:**
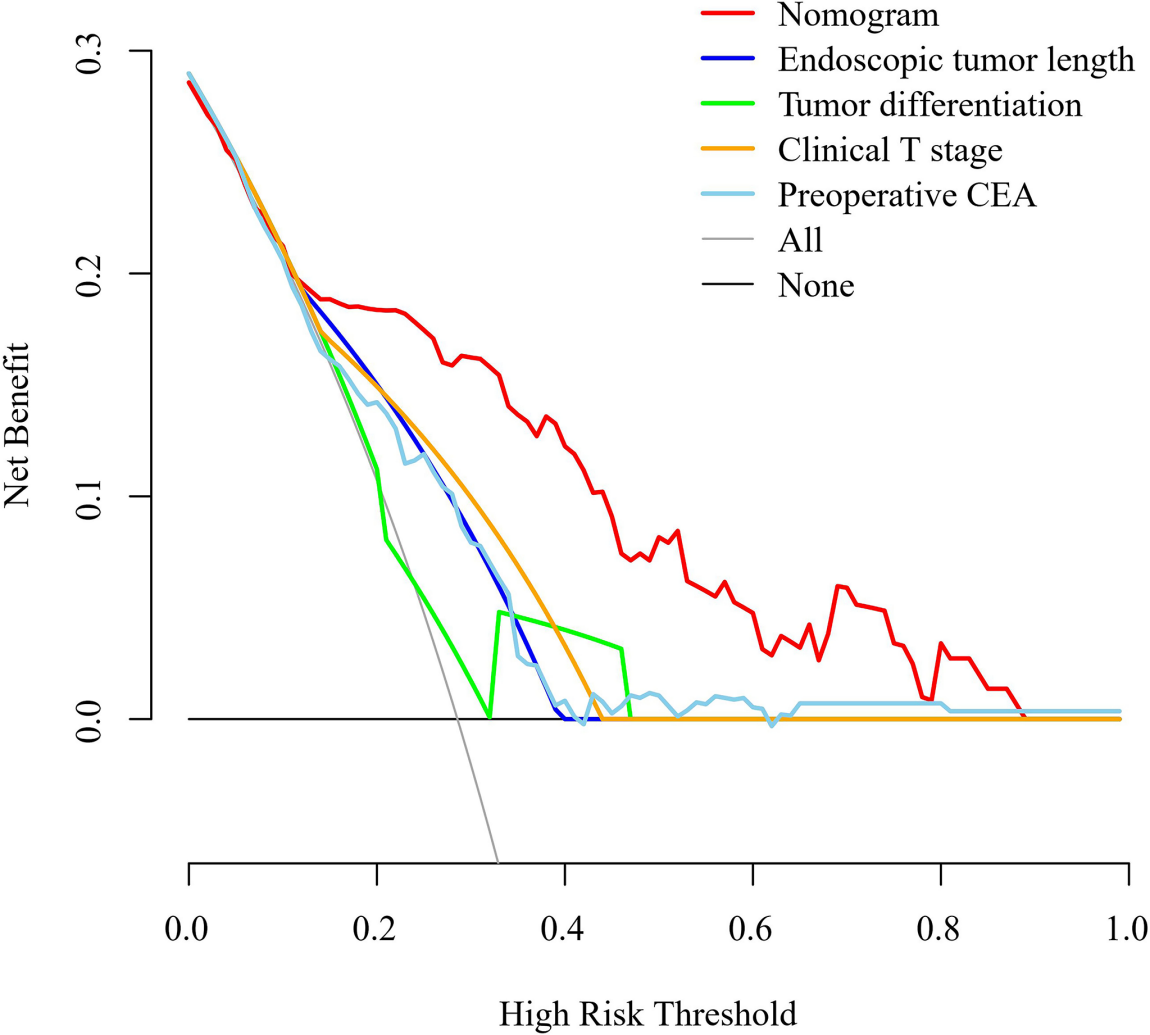
Decision curve analysis for the nomogram, endoscopic tumor length, bioptic tumor differentiation, clinical T stage, and preoperative CEA in the external validation cohort. The clinical T stage and bioptic tumor differentiation were referenced by the 8th edition of the UICC and AJCC cancer staging system. CEA, carcinoembryonic antigen.

A nomogram was developed using the four independent risk factors to estimate the individual risk of RLN LNM (**Figure 4A**). We calculated the total score of four variables involving bioptic tumor differentiation, T stage, endoscopic tumor length, and preoperative CEA, which could be summed by adding each score and projecting it onto the total point scale to identify the predicted probability. The nomogram demonstrated good discrimination with a bootstrapped AUC of 0.770 (95% CI, 0.685–0.850) and good calibration with a bootstrapped Brier score of 0.170 (95% CI, 0.139–0.210). In the external validation, the model showed an excellent performance with an AUC of 0.832 (95% CI, 0.765–0.908) and a Brier score of 0.159 (95% CI, 0.111–0.198) (**Figure 4B**). The nomogram predicted RLN LNM probabilities in the calibration plots were consistent with the actual probabilities in the external validation cohort (**Figure 4C**). DCA plots revealed that compared with endoscopic tumor length, bioptic tumor differentiation, clinical T stage, and preoperative CEA, the nomogram had higher net benefits (**Figure 5**).

### Preoperative Predictors of Survival

**Figure 6:**
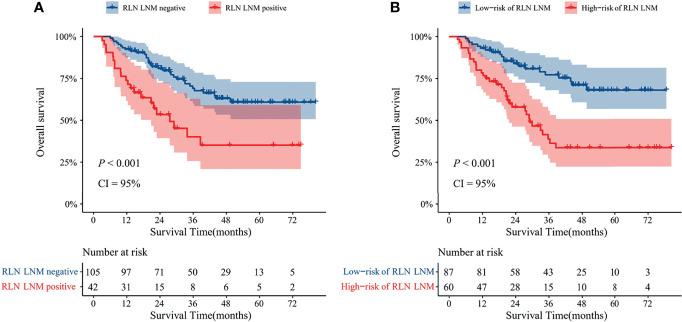
Kaplan–Meier curves with 95% confidence intervals for overall survival of patients stratified according to the RLN LNM status **(A)** and nomogram predicted RLN LNM risk **(B)** in the external validation cohort. RLN LNM, recurrent laryngeal nerve lymph node metastasis, CI, confidence interval.

In the external validation cohort, 60 of 147 patients were classed to high-risk of RLN LNM group by the predicted values with the optimal threshold of 0.471, while 87 of 147 patients to low-risk of RLN LNM group (**Figure 4B**). The mean overall survival for the RLN LNM positive versus negative and nomogram predicted high-risk versus low-risk of RLN LNM in the external validation cohort were 37.8 (95% CI, 27.8–47.8) versus 59.0 (95% CI, 53.1–64.8) and 40.3 (95% CI, 32.0–48.6) versus 61.9 (95% CI, 55.8–68.0), respectively. The prognostic value of RLN LNM risk with HR of 3.208 (95% CI, 1.818–5.662; *P <*0.001) was comparable to that of RLN LNM status with HR of 2.493 (95% CI, 1.408–4.415; *P* = 0.002) after adjusting age, sex, tumor location, hemoglobin, neutrophil, HDL-C, and LDL-C. Patients with RLN LNM positive or high-risk of RLN LNM had a worse overall survival than those who were RLN LNM negative (*P <*0.001, **Figure 6A**) or low-risk of RLN LNM (*P <*0.001, **Figure 6B**) in the external validation cohort.

## Discussion

Considering the significant value of RLN LNs status of patients with ESCC for treatment guidance, we sought to investigate the independent risk factors and construct a preoperative model for RLN LNM and survival prediction. In this study, the following findings were revealed. (a) Clinical T stage, bioptic tumor differentiation, endoscopic tumor length, and preoperative CEA were independent risk factors for RLN LNM. (b) The nomogram developed by the preoperative characteristics could predict the RLN LNs status accurately, which was validated internally and externally. (c) The risk of RLN LNM was significantly associated with the prognosis of patients with ESCC. This nomogram may help to make a more rational clinical treatment decision.

Since the RLN is a common metastasis site, RLN LNs are routinely resected in esophagectomy. However, surgical complications increase RLN injury and permanent RLN palsy with rates of 36 and 12%, respectively (9). Current evidence confirms the effects of RLN LNM on the 5-year survival of ESCC patients at a rate of only 21.7% (23). Other studies also indicated that RLN LNM was a predictor of worse survival and mortality (7, 23, 24). These findings emphasized the necessity of RLN LNM prediction and the balance between extended lymph node dissection and RLN-related complications.

Currently, radiological examinations are usually used to diagnose RLN LNM status before surgery, like CT and positron emission tomography (PET)/CT. In CT images, a diameter of RLN LNM could reflect the status of RLN LNM and short diameter RLN LNs greater than 10 mm are usually judged as metastasis. However, previous studies demonstrated that the cut-off of the short diameter of RLN LNs to diagnose metastasis was shorter than 10 mm, like 6.5 mm (19) and 7.5 mm (6). Additionally, it is well-known that using a CT image to distinguish benign inflammation of LNs from malignant metastasis and to diagnose metastasis in LNs less than 5 mm is difficult. However, a previous study reported that the LNM diameter less than 5 mm accounted for a relatively large proportion (25). These limitations make CT insufficient for preoperative diagnosis of RLN LNM. Although PET/CT is regarded as a more accurate method to diagnose LNM than CT, it faces low sensitivity and is limited in small LNM (26, 27). Therefore, accurate RLN LNM prediction needs to combine with preoperative clinical characteristics of patients with ESCC.

The depth of invasion is important in the staging of esophageal carcinoma. In the different infiltrating stratifications of esophageal carcinoma, there are completely different lymphatic drainage systems, namely, lymphatic vessels in the submucosa containing transverse and longitudinal vessels (28). When the tumor infiltrates the submucosa or deeper, it may further metastasize to the RLN LNs through longitudinal lymphatic vessels (29). Longfei et al. (30) reported that tumor invasion depth significantly influenced RLN LNM. Additionally, almost all relevant previous studies showed that the T stage was associated with RLN LNM (19, 20). In our study, the clinical T stage was regarded as an independent risk factor for RLN LNM, consistent with the current understanding that a higher probability of RLN LNM generally occurs in advanced T-stage ESCC (7).

Existing definite evidence has shown that a poor degree of postoperative tumor differentiation contributes to a high rate of RLN LNM (30, 31). Tumor cells with poor differentiation are similar to immature tissue, where the tumor is highly malignant. In this case, the probability of the transition increases two-fold. Yu et al. (20) reported that well, moderately, and poorly differentiated ESCC had RLN LNM rates of 10.35, 14.15, and 45.46%, respectively. However, our study first introduced bioptic tumor differentiation as one of the potential risk factors which may be more rational for predicting RLN LNM before surgery. In this current study, the well, moderately, and poorly bioptic tumor differentiation of ESCC presented RLN LNM rates of 19.2% (34 of 177), 29.1% (60 of 206), and 63.8% (30 of 47), respectively, which were roughly consistent with previous literature (19, 20).

The present study also found that an endoscopic tumor length more than 3 cm was considered to be related to an increased probability of RLN LNM compared to a smaller tumor. Eloubeidi et al. (32) reported similar findings and suggested that tumor size should be suffixed in the T stage as a modification of the TNM staging system. A previous study reported an optimal cut-off point of 3.5 cm, demonstrating that larger tumors were related to a higher positive LNM, including RLN LNM (33).

In the current study, we found that preoperative CEA level was associated with RLN LNM as shown in the multivariable analysis. However, CEA was reported as having no statistical relationship with RLN LNM in a previous study (20). The reason why it has differed from our result may be that they set CEA as categorical data with a cut-off of 5 ng/ml. Although no specialized literature has confirmed the relationship between CEA and RLN LNM, CEA was shown to be correlated with LNM (34). Tetsuro et al. (35) found that CEA mRNA in the blood is useful for early detection of recurrence in esophageal cancer. Additionally, CEA could be a noninvasive useful diagnostic tool if it can effectively predict RLN LNM since endoscopic ultrasonography, computed tomography, and positron emission tomography cannot exclude RLN LNM completely (36).

Several studies reported the prediction model to estimate the risk of RLN LNM for patients with ESCC. Liu et al. (19) and Yu et al. (20) developed the prediction model for RLN LNM. However, both models are based on the postoperative characteristics of patients with ESCC, which limited the application of this model. Recently, Zhang et al. (6) reported a preoperative nomogram to predict left and right RLN LNM, but their nomogram did not include several critical clinical characteristics like clinical T stage and tumor differentiation. Moreover, all previous studies lacked internal or external validation, which may result in inaccurate performance assessment. In the current study, we included four preoperative risk factors to construct this model. The discrimination, calibration, and clinical usefulness of the model highlighted its performance. Finally, the bootstrap method and hold-out method (external validation cohort) were used to validate this model. The AUCs of our model in the internal and external validation was greater than 0.750, indicating that the nomogram had acceptable discrimination for predicting RLN LNM. Additionally, the predicted probability and actual probability of RLN LNM were roughly equal, and the curve was close to the diagonal in the calibration curve, indicating a good prediction effect. DCA further demonstrated that the nomogram outperformed any single risk factor in predicting RLN LNM in terms of clinical usefulness. This nomogram can be easily applied in clinics with visualization. For example, consider a patient with clinical T1–2 stage (0 points), G3 (22 points), the endoscopic tumor length ≥3 cm (15 points), and the preoperative CEA was 6 ng/ml (30 points). The variables listed above yielded a score of 67 points for this patient, and the corresponding risk of RLN LNM was close to 70% (**Supplementary Figure 1**).

In our study, we further confirmed that RLN LNM was significantly associated with the overall survival of ESCC patients. Longfei et al. (30) reported that patients with RLN LNM had worse overall survival and disease-free survival at any stratification. To the best of our knowledge, our study is the first to assess the prognostic value of the preoperative prediction model of RLN LNM in patients with ESCC. We found that the predicted RLN LNM risk of the nomogram had a comparable prognostic value with RLN LNM, which further demonstrated the great effectiveness of this nomogram to aid in clinical treatment decision making. After adjusting other preoperative factors, the predicted RLN LNM risk is still significantly associated with overall survival. Due to the aim of preoperative prediction, we did not analyze the influence of the adjuvant treatment or other postoperative factors. Theoretically, the patients with high-risk are more likely to diagnose RLN LNM, which may impact the postoperative prognostic factors like adjuvant treatment and further cause a worse overall survival.

## Limitation

The limitations of this current study are presented as follows. First, this study design was retrospective, and there was some inevitable selection bias. A further randomized controlled trial may be performed in our further study. Second, although we performed this study at two centers, the relatively small sample size limited the application of this nomogram. Therefore, investigations with a large sample size are warranted in the future. Third, important endpoints, namely, recurrence and disease-free survival, were not evaluated in this study. However, these endpoints are vital in the outcomes of malignant tumors. We analyzed the overall survival of ESCC patients, which is one of the most important endpoints, and our study can be considered as a starting point for subsequent studies. Fourth, when evaluating the clinical T stage, only limited patients executed PET/CT examination in our current cohort due to high cost. We performed enhanced CT, endoscopic ultrasonography and ultrasonography of the cervical and abdominal region to assess the clinical T stage. Finally, we only included CEA in this study, and other tumor biomarkers may be associated with RLN LNM. Due to limited reports, the tumor biomarkers to predict RLN LNM need further study.

## Conclusion

We constructed a preoperative nomogram that can effectively predict RLN LNM and overall survival with a good performance. Both internal and external validation indicated the great performance of this model. Data from more centers are needed to validate this model further.

## Data Availability Statement

The raw data supporting the conclusions of this article will be made available by the authors, without undue reservation.

## Ethics Statement

The studies involving human participants were reviewed and approved by The Ethics Committees and Review Board of the Affiliated Hospital of North Sichuan Medical College. Written informed consent for participation was not required for this study in accordance with the national legislation and the institutional requirements.

## Author Contributions

Conception, study design and protocol: H-JY, W-JM, R-XY, H-YW, and DT. Data collection and extraction: K-YJ, HH, Z-DZ, and Q-CQ. Methodology assessments: H-JY, W-JM, R-XY, and X-GG. Statistical analyses: H-JY, W-JM, and R-XY. Writing: H-JY, W-JM, and R-XY. Project oversight and supervision: H-YW and DT. Critical revisions for important intellectual content: H-YW and DT. All authors listed have made a substantial, direct, and intellectual contribution to the work and approved it for publication.

## Funding

This research was supported by the Clinical Research Foundation of the Affiliated Hospital of North Sichuan Medical College (2021LC004) (to DT) and Nanchong City-School Cooperation Research Fund (No. 20SXZRKX0001) (to H-YW).

## Conflict of Interest

The authors declare that the research was conducted in the absence of any commercial or financial relationships that could be construed as a potential conflict of interest.

## Publisher’s Note

All claims expressed in this article are solely those of the authors and do not necessarily represent those of their affiliated organizations, or those of the publisher, the editors and the reviewers. Any product that may be evaluated in this article, or claim that may be made by its manufacturer, is not guaranteed or endorsed by the publisher.
