# Supplementary material for: Preoperative Clinical Characteristics Predict Recurrent Laryngeal Nerve Lymph Node Metastasis and Overall Survival in Esophageal Squamous Cell Carcinoma: A Retrospective Study With External Validation
Source: Front Oncol. 2022 Mar 31;12:859952. doi: 10.3389/fonc.2022.859952 (PMC9008727; doi:10.3389/fonc.2022.859952)
Supplement: Supplementary file 1 [file DataSheet_1.docx]

**Supplementary Table 1. Clinical characteristics of esophageal squamous cell carcinoma patients in the entire cohort [RLN LNM (-) versus RLN-LNM (+)].**

| **Characteristics** | **All patients**  **(n=430)** | **RLN-LNM (-)**  **(n=306)** | **RLN-LNM (+)**  **(n=124)** | **P value** |
| --- | --- | --- | --- | --- |
| Age |  |  |  | 0.732 |
| <60y | 113 (26.3%) | 79 (25.8%) | 34 (27.4%) |  |
| ≥60y | 317 (73.7%) | 227 (74.2%) | 90 (72.6%) |  |
| Sex |  |  |  | 0.679 |
| Male | 306 (71.2%) | 216 (70.6%) | 90 (72.6%) |  |
| Female | 124 (28.8%) | 90 (29.4%) | 34 (27.4%) |  |
| Endoscopic tumor length |  |  |  | <0.001* |
| <3 cm | 149 (34.7%) | 131 (42.8%) | 18 (14.5%) |  |
| ≥3 cm | 281 (65.3%) | 175 (57.2%) | 106 (85.5%) |  |
| Tumor location |  |  |  | 0.266 |
| Upper | 78 (18.1%) | 50 (16.4%) | 28 (22.6%) |  |
| Middle | 241 (56.0%) | 173 (56.5%) | 68 (54.8%) |  |
| Lower | 111 (25.9%) | 83 (27.1%) | 28 (22.6%) |  |
| Bioptic tumor differentiation ^†^ |  |  |  | <0.001* |
| G1 | 177 (41.2%) | 143 (46.7%) | 34 (27.4%) |  |
| G2 | 206 (47.9%) | 146 (47.7%) | 60 (48.4%) |  |
| G3 | 47 (10.9%) | 17 (5.6%) | 30 (24.2%) |  |
| Clinical T stage ^†^ |  |  |  | <0.001* |
| T1/T2 | 189 (44.0%) | 164 (53.6%) | 25 (20.2%) |  |
| T3/ T4 | 241 (56.0%) | 142 (46.4%) | 99 (79.8%) |  |
| Hemoglobin (g/dl) | 130.65 ± 15.31 | 130.46 ± 15.85 | 131.12 ± 13.93 | 0.684 |
| Neutrophil (10^9^/L) | 4.03 ± 2.05 | 3.95 ± 1.94 | 4.22 ± 2.30 | 0.428 |
| Preoperative CEA (ng/mL) | 5.10 ± 1.57 | 4.84 ± 1.33 | 5.74 ± 1.91 | <0.001* |
| HDC-L (mmol/L) | 1.31 ± 0.34 | 1.32 ± 0.35 | 1.28 ± 0.29 | 0.274 |
| LDL-C (mmol/L) | 2.94 ± 0.72 | 2.91 ± 0.70 | 3.00 ± 0.76 | 0.245 |

*: P＜0.05; RLN LNM, recurrent laryngeal nerve lymph node metastasis; ^†^ The 8th edition of the UICC and AJCC cancer staging system; CEA: carcinoembryonic antigen; HDL-C: high density lipoprotein-cholesterol; LDL-C: low density lipoprotein-cholesterol.


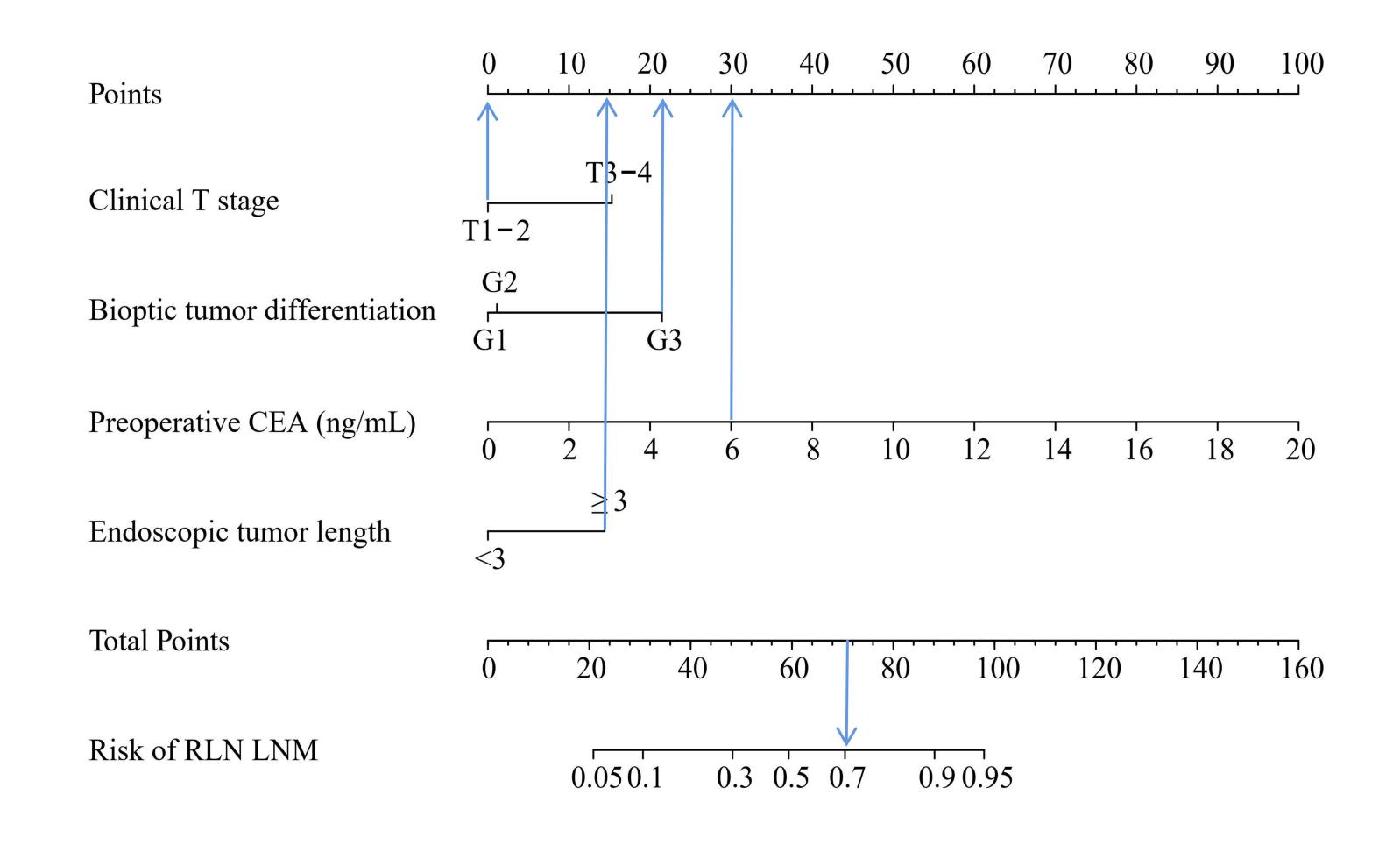


**Supplementary Figure 1**. Application of this nomogram. The total score is 67 (0+22+15+30) and the corresponding risk of RLN LNM is about 70%. CEA: carcinoembryonic antigen; RLN LNM: recurrent laryngeal nerve lymph node metastasis.
